# The impact of the livelihoods and income fortification and socio-civic transformation project on the quality of life, wellbeing, self-esteem, and quality of neighbourhood social environment among the youth in slum areas of in Kampala, Uganda

**DOI:** 10.1186/s12889-020-09868-y

**Published:** 2020-12-07

**Authors:** Andre M. N. Renzaho, Daniel Doh, Rashidul A. Mahumud, Moses Galukande, Joseph K. Kamara

**Affiliations:** 1 School of Social Sciences, Western Sydney University, Penrith, 2751 Australia; 2 Translational Health Research Institute, Western Sydney University, Penrith, 2751 Australia; 3 Maternal, Child and Adolescent Health Program, Burnet Institute, Melbourne, 3004 Australia; 4 Makerere University College of Health Sciences, P.O. Box 7072, Kampala, Uganda; 5 World Vision International, East Africa Regional Office, Karen, Nairobi, Kenya

**Keywords:** Quality of life, Young people, Youth, Uganda, Slums, Well-being, Self-esteem

## Abstract

**Background:**

Various interventions to improve the quality of life (QoL) among slum dwellers across sub Saharan Africa have been implemented. However, the interventions impacts remain less understood. We assessed the impact of the Urban Program on Livelihoods and Income Fortification and Socio-civic Transformation (UPLIFT) project on QoL, psychological wellbeing, self-esteem, and the quality of neighbourhood social environment of young people aged 13–25 years in slum areas of Makindye and Nakawa Divisions in Kampala, Uganda.

**Methods:**

The study was designed as a mixed method evaluation using repeated cross-sectional survey and grounded theory in both the intervention and comparison communities. The intervention effect was estimated using the difference-in-differences Kernel propensity-score matching technique, with bootstrapping. The “rcs” option was used given that data were from repeated cross-sectional surveys. A thematic analysis was adopted for the qualitative data to triangulate and complement the quantitative data.

**Results:**

The UPLIFT project led to an improvement in QoL, psychological wellbeing, and self-esteem of young people. In terms of QoL, the project led to a six-percentage point increase in quality of living conditions scores (where higher scores reflect better living conditions; lower ones, worse living conditions). However, a negative effect was observed for personal independence whilst the project did not have any impact on social relations. In terms of self-esteem and psychological wellbeing, the project led to a 4.6-point increase in self-esteem scores, a 5.4-point increase in self-acceptance scores, a 5.3- point increase in purpose in life scores, a 5.7 - point increase in personal growth, and a 10.7-point increase in autonomy scores. However, the project had a negative effect on personal independence; and had no impact on environmental mastery and the quality of neighbourhood social environment.

**Conclusion:**

Functional community-owned assets accumulation and capacity building initiatives for young people in slum areas improved their psychological wellbeing and quality of life. However, such initiatives do not appear to address social relationships and personal independence of young people in slum areas.

## Background

Slums are unhealthy, unsafe, and socially undesirable residential areas characterised by substandard housing and living conditions as well as overcrowding [1]. Slums characterise poverty and inequality in low and middle income countries (LMICs), with a common thread in such a hodgepodge of poor housing structures being the lack of one or more of the following five amenities*:* (1) durable housing capable of withstanding extreme climatic conditions; (2) sufficient living area commensurate with the needs of family members and the community they are part of; (3) access to improved and affordable water sources and quality; (4) access to improved sanitation facilities; and (5) secure tenure that protects against forced eviction [1].

The United Nations Statistics Division indicate that in 2018 more than one billion people (1.033 billion) lived in slums globally, and by 2030 an estimated 3 billion people will require adequate and affordable housing [2]. Whilst the health of slum dwellers is poorly documented, emerging evidence suggests that they have poorer physical and mental health outcomes than non-slum dwellers, including having poorer quality of life (QoL) and psychological wellbeing; having a higher burden of diseases, and adopting unhealthier lifestyles and riskier health behaviours [3–7].

Nonetheless, the last two decades have seen tremendous global efforts to improve the living conditions among slum dwellers, resulting in a remarkable reduction in slum population by 23% between 1996 to 2016 across LMICs [8]. This reduction was a result of concerted efforts of the United Nations’ commitment to improve the QoL of slum dwellers under target 7 of the Millennium Development Goals (MDGs) and goal 11 of the Sustainable Development Goals (SDGs). Target 7d of the MDGs committed to improve the lives of 100 Million slum dwellers [9]. Goal 11 of the SDGs commits to making cities and human settlements inclusive, safe, resilient, and sustainable with a specific indicator to monitor slum populations [8, 10]. Despite the global efforts, slum dwelling continues to hamstring the QoL and the psychological wellbeing of poor urban populations in MLMCs.

Sub Saharan Africa has a high prevalence of slums accentuated by high population growth unmatched by the weak economic opportunities, rapid rural to urban migration, and weak urbanisation strategies. The countries with the largest proportion of urban populations in slum conditions across the region include Central African Republic (96%), Chad (89%), and Mozambique (81%). Others are: Niger, (82%) Ethiopia (76%), Somalia (74%), Tanzania (64%) and Uganda (60%) [11], where people live in poor conditions.

Some of the largest slums in the region include: Khayelitsha in Cape Town with a population of over 400,000; Kibera (also known as Kibra) in Nairobi, which hosts a population of nearly 186,000 people [12]; Ashaiman in Tema, Ghana which hosts over 150,000 people and the slums of Makindye, and Nakawa Divisions of Kampala in Uganda. Makindye Division has a population of almost 400,000 people; the majority of the population dwell in the slums adjacent to the affluent areas of Kampala Central [13]. Similarly, Nakawa division hosts a population of 317,023, the majority of these live in slums with poor services, for example, almost 16 and 27% of the households live 5 km or more to the nearest public health facility [14]. Makindye and Nakawa slums provide low cost accommodation for many young rural-urban migrants search of city opportunities.

There have been multiple attempts by the Ugandan Government to improve young people’s QoL through availing employment and employability opportunities, entrepreneurial skills and wealth creation. Such efforts include: Operation Wealth Creation (OWC) programme led by the Ugandan military and aimed to transform subsistence to commercial farming among youth and to increase rural opportunities while discouraging rural to urban migration [15]; Skilling Uganda program aimed at mass increase of youth attainment of practical skills to promote employability [16]; National Agricultural Advisory Services (NAADS) which supports agricultural input distribution, value chain development and farmer access to agricultural financing [17]; Savings and Credit Co-operatives (SACCOs) which were hyped as inclusive mechanisms to transform Uganda into a middle-income nation by 2020 [18]. The efforts were well intended but have not successfully yielded the desired results mainly because of various encumbrances. For example, the efforts were politically orchestrated without scientific evidence and lacked the critical mass to transform the country’s largest population segment [17–19].

There is limited evidence of effectiveness of interventions on QoL among slum dwellers. A Cochrane review examined the effect of slum upgrading on slum dwellers’ health, QoL and social wellbeing [4]. It found that, overall, slum upgrading led to a reduction in diarrhoeal diseases and water-related expenses. However, there were mixed outcomes for the intervention’s impact on general measures of communicable diseases, financial poverty, and unemployment. The authors of the study concluded that future research with robust study designs and tested common outcome measures is needed to examine how best to improve the QoL, health and psychological wellbeing, social, and economic outcomes of dwellers slum. Therefore, the current study sought to fill in this gap and examined the impact of the Urban Program on Livelihoods and Income Fortification and Socio-civic Transformation (UPLIFT) on the quality of life, psychological wellbeing, self-esteem, and the quality of neighbourhood social environment of young people aged 13–25 years in slum areas of Makindye and Nakawa Divisions in Kampala, capital and largest city of Uganda.

## Methods

### Setting and description of the intervention

More than two thirds (> 67%) of Kampala’s population is made up of unemployed or under-employed youth, many of whom resort to crime, gambling, drug abuse, and other social problems for survival. Many of the youth also live in slum areas [20, 21]. Uganda, as is the case in most African countries, attracts the youth to the city of Kampala due to perceived opportunities and better life in urban areas. It is estimated that in the case of Kampala, 60% of its population live in slum conditions [22]. Administratively, Kampala is divided in five divisions which are further subdivided into parishes made of clusters of villages [23, 24]. The UPLIFT project was a four-year project (2014–2017) that aimed to improve the QoL of young people aged 13–25 years in slum areas of two main city divisions of Makindye and Nakawa. The two divisions have parishes with the highest concentration of unemployed, under-employed, and out of school youth. Therefore, UPLIFT project targeted the most disadvantaged parishes in these divisions.

The project had six components: job markets and employment, sexual and reproductive health rights (SRHR), child protection, civic engagement, support and advocacy for young people with disability, and existing policy and law enforcement (e.g. raising community awareness of available laws on risky social behaviours). The emphasis was on increasing employment potential/employability of young people, access to sexual and reproductive health rights information and services, protection against abuse and exploitation, and increasing level of civic awareness among most vulnerable young people aged 13–25 years and their families. **Tables** **1** and **2** summarise the intervention components and the project’s key components and structure. The project was funded by the Australian government and implemented by World Vision Uganda, in partnership with Uganda Youth Development Link, Uganda Youth Network, and Kampala City Authority (KCCA).

**Table 1:** Description of the intervention

Intervention domain	Activities carried out	Target populations	Number of sessions/outputs	Frequency of delivery	Agency responsible
**Job markets and employment**	✓ Vocational skills training	Out-of-school 16–25 year olds	496 youths trained	4 months training with internship	World Vision Uganda, Lugogo Vocational Training Institute, KCCA
	✓ Saving group formation and training	All young people aged 13–25 years	1873 members, USD71,429 in cumulative savings 75 groups registered as Community-Based Organisations 3 groups have accessed KCCA Community Development Driven grant	Weekly saving group meetings	World Vision Uganda
	✓ Information sessions between Business Savings and Loan Associations (BSLA) groups and financial service providers and facilitating opening of bank accounts,	BSLA members, financial institutions	75 BSLAs opened up group accounts in banks. 1000 members attended bank exhibitions and accessed financial literacy education	Once a year in each division	World Vision Uganda, KCCA, Development Finance Company of Uganda (DFCU) bank, Centenary and Post Banks.
	✓ Saving group meetings and Community Based Trainers (CBT) training and mentorship	13–25 year olds			World Vision Uganda
	✓ Business and entrepreneurship skills training	BSLA members, CBTs and vocational skills graduates	2379 members trained	Once every year	World Vision Uganda
	✓ Mentorship groups	BSLA members	1873 members attached to a community business mentor	Once every month	World Vision Uganda
**Sexual and reproductive health needs and rights**	✓ Peer-to-peer SRHR sessions in community and youth centres.	13–25 year olds in both divisions	A total of 30 peer-to-peer sessions were conducted in the community; 641 young people (205 male, 436 female) reached with SRH/HIV information	One session per peer education every month	Uganda Youth Development Link, Religious Institutions, KCCA
	✓ Community outreaches and referrals	13–25 year olds in both divisions	4796 youths accessed youth-friendly SRHR services	Outreaches conducted 2 times every week	Uganda Youth Development Link, KCCA
	✓ Tracking complete referrals from community outreaches to health centres	13–25 year olds	A total of 325 cases of young people (57 male, 268 female) referred during this period. 271young people (41 male, 230 female) tracked as complete referrals to various health centres.	Every month	Uganda Youth Development Link, China Friendship Hospital, Kisugu Health Centre, Kiswa/Naguru Health Centre, KCCA, Kisenyi Health Centre
	✓ Learning visits by youths to SRH sites	Young people rehabilitated at the youth centres	Eight exposure learning visits conducted. 183 young people (69 male, 114 female) participated in the visits at friendly SRH sites/ facilities	Once every quarter for four years	Uganda Youth Development Link, KCCA health centres
	✓ Meetings with religious leaders to combat sex work among youths	Religious leaders from different religious affiliations	57 religious leaders (representing 45 religious institutions) were equipped with SRH information mobilising the community and youths involved in commercial sex to seek these services.	Once a quarter	Uganda Youth Development Link
	✓				
	✓ SRHR life skills training for 20 youth clubs at each religious institution;	Youths subscribing to youth clubs formed at the religious institutions	400 youths received life skills training	Every month	20 religious institutions, Uganda Youth Development Link
	✓ Feedback meetings on progress of SRHR interventions	All youths in six targeted parishes in Nakawa and Makindye	4 feedback meetings held	Once every year	Uganda Youth Development Link
	✓ Safe male circumcision	Males aged 13–25 years	498 boys circumcised	Conducted Feb–Apr 2016	Uganda Youth Development Link, KCCA health centres, World Vision Uganda
**Child protection**	✓ Linkage meetings between formal and informal child protection structures	All child protection structures (police, child protection committees, judiciary, probation, school teachers, parents/caretakers)	12 meetings	Once every quarter	World Vision Uganda, KCCA
	✓ Dialogue meetings	All community members in the 6 F	72 dialogue meetings	Every quarter	World Vision Uganda, Probation office
	✓ Inter-talent events for schools, child rights club activities	10 primary schools	1 talent event	Once	10 primary schools, World Vision Uganda
	✓ Refresher training for Child Protection Committees (CPCs) on record keeping	CPCs and teachers	120 CPCs and 20 teachers oriented on record keeping in 2016	Once	World Vision Uganda, KCCA
	✓ Psychosocial support training	Child protection committees and teachers	1 training session conducted	Once	World Vision Uganda
	✓ Community gatherings to address child protection issues	All community members	48 gatherings conducted	Once a quarter	World Vision Uganda
	✓ Case management by formal and informal structures	CPCs and households with child protection issues	368 cases handled	Ongoing case by case	KCCA, police, World Vision Uganda
	✓ Monitor informal child protection structures to ensure that they are functioning	Child protection committees	2 monitoring and support visits done	2 times a year	World Vision Uganda, KCCA, police
**Civic engagement**					
	✓ Selected youth groups supported to continuously assess the different service delivery in different public institutions	Youths aged 16–25 years in Civic Voice and Action working team	25 youths in working teams supported to monitor service delivery	Every month	Uganda Youth Network, KCCA, World Vision Uganda
	✓ High-level dialogue engagements with KCCA to develop divisional and joint advocacy plans	KCCA technical and political teams	2 high level dialogue engagements with 506 young people held	Once in 2016 and 2017	Uganda Youth Network, KCCA, World Vision Uganda
	✓ Community dialogues between youths and community leaders to discuss issues affecting youth	Youth and community members	38 dialogues	Every month	Uganda Youth Network, KCCA,
	✓ Support the CVA groups to hold 8 community gatherings for improved service delivery	Community members	8 gatherings	Bi annual	Uganda Youth Network, Youth CBOs
**Disability**	✓ Advocate for people with disability	Young people (0–18) with disability	75 given wheelchairs	One-off activity	World Vision Uganda, KCCA
**Existing policy and law enforcement**	✓ Raise community awareness of available laws on risky social behaviours	Entertainment centres, community members, local leaders	12 meetings held	Quarterly for 3 years	KCCA, Uganda Youth Network, Uganda Youth Development Link

**Table 2:** Project theory of change, components and structure

**Project Goal:** To reduce the proportion of young people aged 13–25 years in Makindye and Nakawa divisions with low quality of life from 42.7 to 30% by 2017	
***Summary of Outcomes:***	
**Outcome 1:** Increased employment potential of young people aged 16–25 years in Makindye and Nakawa Divisions by 2017	
***Output 1.1:*** Access to credit and savings opportunities for young people in Nakawa and Makindye Divisions increased	
***Output 1.2:*** Young people equipped with Vocational Skills for self-employment	
***Output 1.3:*** Young people empowered with Business and Entrepreneurship skills	
**Outcome 2:** Increased access to SRHR information and services among 3500 young people aged13–25 years in Makindye and Nakawa Divisions of Kampala City by 2017	
***Output 2.1:*** Sexual and Reproductive Health Services at Community and Health Facilities strengthened.	
***Output 2.2:*** Capacity of service providers to provide Youth Friendly SRHR enhanced	
***Output 2.3:*** Exposure and risk to alcohol, drug and substance abuse reduced	
**Outcome 3:** Increased protection against abuse and exploitation among young people aged 13–25 years in Nakawa and Makindye Divisions by 2017	
***Output 3.1:*** Community Based Child Protection Formal and Informal mechanisms are functional	
***Output 3.2:*** Community awareness on Child Wellbeing including child rights, participation and child protection increased	
**Outcome 4:** Increased involvement of young people in dialogue with government and other actors on development.	
***Output 4.1:*** Capacity of young people aged between 13 and 25 enhanced to engage in civic activities for improved service delivery	
***Output 4.2***: Young people empowered with Life Skills to claim for their rights	
***Output 4.3:*** Young People effectively Engaging with duty bearers for improved service delivery	

### Study design and sampling strategies

Using a repeated cross-sectional design, qualitative and quantitative data were simultaneously collected in July 2014 and June 2017. The mixed method design allowed the study team to triangulate emerging findings and to generate rich contextual information as well as provide a comprehensive picture of the project’s impact.

The repeated cross sectional data were obtained from parishes in Makindye and Nakawa divisions. Makindye division is made of 21 parishes and 884 villages, while Nakawa division is made of 23 parishes, 669 villages. Although the UPLIFT intervention engaged grassroots community organisations and worked closely with the Kampala City Council Authority (KCCA, coordination of activities with other non-governmental organisations (NGOs) was limited. Of the 44 Parishes, 26 met the project criteria, of which 6 were outside the project’s area boundaries and/or were receiving assistance of similar nature from other NGOs. There were cases where, in Nakawa division for example, three NGOs withdrew from the activity to prevent duplication and moved to other parishes. Hence, of the 20 parishes meeting the project’s inclusion criteria and within the project’s area boundaries, sixteen (80%) were selected for inclusion in the study. Six parishes among those that met the inclusion criteria fell outside the project’s area boundaries and only two (2/6 = 33.3%) were not contaminated (2/6 = 33.3%); they were not exposed to the project or receiving similar activities from other NGOs and were included as comparators. A list of all villages within the parishes covered by the intervention and parishes not served by the project was compiled with the assistance of local councils and World Vision Uganda. Households sampled were selected using a systematic sampling approach and the probability proportional to the household size sampling was utilised to account for varying village sizes. The total number of households in each village was divided by the expected sample size to obtain the sampling interval (S). We considered a unique identification number we gave to each household in each village to determine the first household to be surveyed by randomly selecting a number between 1 and S. Subsequent households were selected by first adding S to the first randomly selected number, and the process continued until we reached the required sample size. Included households were those with eligible participants aged 13–25 years. This was the age group targeted by the intervention. When the household was selected, the head of the household was asked whether any member of the family was aged between 13 and 25 years. Thereafter, eligible participants were invited to voluntarily partake in the study within their homestead environment. Participants who accepted the invitation were afforded privacy so as to answer the questions truthfully. Hence, the interview took place away from other family members to prevent eavesdropping. In cases where the selected household was inhabited, had no one at home at the time of the survey, or did not have an eligible participant, the closest neighbouring household was selected for the survey. For household with more than one eligible participant, the interviewer randomly selected one participant to be included in the study.

Qualitative respondents were purposively sampled using different strategies: geographically (according to their slum location), by affiliation and membership to any community organisation, and through places of worship, sport venues, and schools. They were recruited with the assistance of youth and community leaders and other organisations working with youth. The key informant interview (KIIs) participants were youth leaders, youth workers, a KCCA official, religious leaders and sports betting business administrators. Focus group discussion (FGD) participants were drawn from young people in sports betting, religious and community leaders, youth in mechanic and metal fabrication workshops, women groups, village saving and loan associations (VSLA), civic voice and action (CVA) groups, community-based youth trainers, and primary carers/ care takers.

### Data collection instruments and procedures

Our data collection questionnaire was based on previously validated instrument which had four areas: QoL; psychological wellbeing, self-esteem, the quality of neighbourhood social environment [25].The QoL instrument comprised 36 items, which we validated in previous studies using structural equation modelling and were found to have robust psychometric properties [19]. The QoL instrument had three sub-scales: social relations with internal consistency measured by a Cronbach’s alpha (α) of 0.86, living conditions (α = 0.84), and personal independence (α = 0.76).

Psychological wellbeing was measured based on Ryff and Keyes scale [26], which incorporates 6 key areas: self-acceptance (young people’s accurate perception of their actions, motivations, and feelings); personal growth (young people’s dedication to realising their potential, and to continued growth); purpose in life (young people’s ability to set goals and define objectives to give life direction in a purposeful & meaningful way); positive relationships (having satisfying relationships with other people through mutual trust and empathy); environmental mastery (young people’s ability to choose or create environment that enable goals and needs to be met); and autonomy (young people’s ability to maintain independence and individuality in different contexts, and to resist social pressure) [26, 27]. Item scores ranged from 1 = strongly disagree to 5 = strongly agree. The scale has been translated and validated in many languages and used across the lifespan and settings [26–29]. In our study, the scale had adequate internal consistency: α =0.716 for autonomy, α =0.749 for environmental mastery, α =0.714 for growth, α =0.793 for positive relations, and α =0.736 for purpose in life.

Self-esteem was measured using the Rosenberg self-esteem scale [30], which contains 10 statements measuring various aspects of self-worth. Item scores ranged from 0 = strongly disagree to 3 = strongly agree. It has been translated and validated in many languages and applied in many settings [31]. In our study, the Rosenberg self-esteem scale had adequate internal consistency, with an α =0.717.

Quality of neighbourhood social environment was measured by three questions. The first question asked participants about drugs and substance abuse as follows: “Over the past four weeks, have you used any of the following substances: khat, marijuana, heroin, cocaine, sedatives, paint sniffing, petrol sniffing, glue sniffing, cigarette, stimulants, kuber (oral tobacco) or shisha?” Any positive response to one or more of these substances was recoded as drug and substance abuse. The second question measured gambling activities using a one item screen as follows: “Over the last 12 months, have you gambled or been involved in betting activities?” This one item screen has been shown to have acceptable performance when compared to the 9-item Problem Gambling Severity Index [32]. The last question asked participants whether they felt safe in their neighbourhood (where 1 = Feel safe MOST of time, 2 = Feel safe SOME of time, and 3 = I don’t feel safe).

For the qualitative component, data collection used KII guide and FGD schedule, informed by the grounded theory. The application of the grounded theory allowed us to explore real-life situations in slums, understand actions and strategies used by young people to navigate challenges and opportunities brought about by their slum environments, and depict the impact of the project on their situation [33]. The objective of using grounded theory was to triangulate data from the surveys through data saturation, whereby rich and in-depth data on young people’s framing of their experiences and the project impact was obtained.

Written and or verbal consent (witnessed) was obtained from participants prior to their participation in data collection. For younger participants (13–15 years), representing 17% of the sample, parental consent was not sought for many reasons. The study areas have a significant of number of slum dwellers, many from rural areas searching for jobs in Kampala, the capital city. They do not live with family members, and there is no way of establishing a valid parental consent for participation. Given the difficulty with obtaining parental consent, we relied on young people’ ability to consent. Due to these challenges, many studies among the slum areas of Kampala, approved by various Institutional Review Boards, have postulated that those who cater for their own livelihood far away from their parents are emancipated and are able to provide their own consent without parental consent [34–37]. In addition, current guidelines on reducing age-related barriers to sexual health services recommend that no parental consent is required when obtaining data on testing, treatment and care, and access to and utilisation of sexual and reproductive health and rights services [38, 39]. Addressing young people’s sexual and reproductive health and rights was the thrust of the UPLIFT project. Parental consent would have negated young people’s freedom to discuss their drug use and experiences with gambling and HIV and sexual health services.

Data were collected by 18 (Male = 9; Female = 9) trained enumerators (14 for the youth surveys) and (4 for the qualitative components) supervised by four experienced field coordinators to monitor quality control. Data collectors were either fresh university graduates or employees of organisations working with youth in urban areas. The training in data collection proceedures lasted two days, followed by a field testing of the questionnaire prior to data collection to ascertain its cultural appropriateness.

Despite the intention of development programs to transform the lives of people, calculating a sample size for evaluating their impact remains a challenge for many reasons. Donors usually do not allow sufficient funds for program evaluations and most NGOs do not allocate enough resources for community engagement and project outreach due to limited access to and time available for adequate project monitoring [40–42]. Such a challenge is compounded by lack of staff’s technical skills and limited access to affordable external sources for timely technical expertise in intervention evaluation [41, 42]. For these reasons, an independent sample was selected at each project phase (baseline and follow-up), and reliable estimates were possible, provided the sample size was adequate at each project phase [43]. Therefore, given that the main challenges for youth living in slums was unemployment, the sample size was calculated using unemployment rates. Estimating the rate of unemployment among youth remains a challenge as 90% of the youth were employed in the informal sector. However, official data indicate that the national unemployment rate is 9.2%, while the unemployment rate for youth aged 18–30 is 18.6% [44]. Such figures do not reflect the plight of young people as they join the workforce at a very young age, often performing insecure, dangerous and non-rewarding jobs that do not offer any decent income. To account for these discrepancies for the sample size calculation at baseline, the study considered an unemployment rate of 50% [45], a 95% confidence interval (CI), a power of 80%, a desired precision level of 3%, and design effect of 2. It was estimated that 600 young people would need to be surveyed. Adjusting a 10% non-response rate gave a total sample of 660 participants. The project covered 80% of the parishes with the highest unemployment rates, hence the sample reflected this distribution. For the pre-intervention survey, data were obtained on 663 (parishes covered by the project = 512, parishes not covered by the project = 151) young people. These similar parameters were used at the follow-up survey but an unemployment rate of 45% was used as the unemployment rate was assumed to have dropped during the second survey, giving a final sample size of 523 participants. For the follow-up survey, data were obtained on 559 (intervention = 456, control group = 123) participants. For the qualitative component, data were obtained from 20 and 13 KIIs during pre and post intervention surveys. Data were also obtained from 10 and 13 FGDs at pre and post intervention surveys. Data collection was governed by a steering committee which approved all research processes to maximise cultural appropriateness.

### Statistical analyses

Data were analysed using STATA version 14 (StataCorp, College Station, TX, USA). Descriptive statistics were used to summarise key variables. Given the cross-sectional nature of the repeated surveys, propensity scores were used in difference-in-differences models to improve covariate balance [46–48]. A logit model was used to estimate program participation (probability of being or not being in the intervention) as a function of the young person’s age, gender, living structure, marital status, and employment status. We then used the predicted values from logit to generate propensity scores for all households in the intervention and comparison groups, and the covariate balance was satisfactory (Table 3). The intervention effect was estimated using the difference-in-differences Kernel propensity-score matching, with the “rcs” option given that our data are from a repeated cross-section design. The “test” option combined with kernel was performed for the balancing t-test with the weighted covariates [49].

**Table 3:** Characteristic of participants at baseline and follow-up; and estimate of program participation

Matching Variables	Intervention				Comparison				Program participation: Logit Model			
	Baseline		Follow-up		Baseline		Follow-up					
	N	%	N	%	N	%	N	%	Coefficient	95%CI	*p*-Value	
**Age in years (Mean ± sd)**	506	19.6 (3.5)	456	19.2 (3.0)	151	19.5 (3.6)	123	19.4(2.9)	0.03	−0.03	0.09	0.310
**Education in years (Mean ± sd)**	506	9.1 (4.0)	456	11.0 (3.1)	151	9.3 (4.0)	123	11.5 (2.7)	−0.04	− 0.09	0.01	0.098
**Gender**	504	100.00	456	100.00	146	100.00	123	100.00				
Female	220	43.65	224	49.12	72	49.32	58	47.15	**Ref**			
Male	284	56.35	232	50.88	74	50.68	65	52.85	0.10	−0.19	0.39	0.506
**Living structure (%)**	471	100.00	456	100.00	139	100.00	123	100.00				
Live with in a nuclear family	128	27.18	112	24.56	24	17.27	29	23.58	**Ref**			
Live with single parent family	107	22.72	87	19.08	43	30.94	15	12.20	−0.27	−0.70	0.17	0.227
Run away	105	22.29	114	25.00	35	25.18	43	34.96	−0.64	−1.11	− 0.18	0.007
Live with other family members	102	21.66	108	23.68	23	16.55	23	18.70	0.05	−0.41	0.51	0.819
Live with non-family members	29	6.16	35	7.68	14	10.07	13	10.57	−0.71	−1.30	−0.12	0.019
**Marital status (%)**	504	100.00	456	100.00	146	100.00	123	100.00				
Single	409	81.15	395	86.62	119	81.51	104	84.55	**Ref**			
Married/Cohabitation	76	15.08	59	12.94	21	14.38	19	15.45	0.22	−0.26	0.71	0.363
Divorced	19	3.77	2	0.44	6	4.11	0	0.00	−0.32	−1.32	0.68	0.532
**Employment status (%)**	503	100.00	456	100.00	147	100.00	123	100.00				
Employed (on a salary)	111	22.07	71	15.57	21	14.29	20	16.26	**Ref**			
Self-employed	129	25.65	140	30.70	36	24.49	36	29.27	−0.01	−0.46	0.44	0.959
Still at school (Student)	148	29.42	141	30.92	42	28.57	27	21.95	−0.03	−0.52	0.46	0.905
Unemployed	115	22.86	104	22.81	48	32.65	40	32.52	−0.55	−0.99	−0.10	0.016

To address variability of estimated program effect [50], bootstrap methods were used by drawing bootstrap samples from the matched pairs in the propensity-score-matched sample [50]. This method resulted in improved estimates of the standard error, [50]. In all cases *p* < 0.05 was considered to be statistically significant.

## Results

### The impact of UPLIFT on QoL

The quality of life measure as demonstrated in this paper comprises the variables: social relations, living conditions, and personal independence. The difference-in-difference estimation (See Table 4) shows that the UPLIFT project has led to [adjusted mean difference-in-differences (standard error)] a six percentage points increase in living conditions [DiD =5.60 (1.77), *p* < 0.01]. However, a negative effect was observed for personal independence [DiD = − 5.22 (1.77), p < 0.01]; there was no observable intervention impact on social relations (DiD = − 1.43 (1.80), *p* = 0.430).

**Table 4:** Program impact on household QoL outcomes

	Baseline			Follow-up			UDiD^b^		ADiD^ba^	
	*Control*	*Intervention*	*Diff (SE)*	*Control*	*Intervention*	*Diff (SE)*	Mean (SE)	*p-value*	Mean (SE)	*p-value*
**Psychological wellbeing**										
Autonomy	22.75	18.09	−4.67 (0.36)	20.16	26.21	6.06 (0.24)	10.73 (0.43)	0.000	10.73 (0.40)	0.000
Environmental mastery	22.57	22.58	0.01 (0.34)	20.14	19.43	−0.71 (0.21)	−0.72 (0.42)	0.088	−0.72 (0.49)	0.141
Personal growth	22.90	20.87	−2.04 (0.34)	24.00	27.62	3.62 (0.32)	5.65 (0.49)	0.000	5.65 (0.50)	0.000
Positive relationships	20.86	22.09	1.23 (0.39)	25.26	21.06	−4.20 (0.25)	−5.43 (0.47)	0.000	−5.43 (0.44)	0.000
Purpose in life	22.49	20.55	−1.93 (0.44)	24.13	27.53	3.40 (0.33)	5.33 (0.56)	0.000	5.33 (0.45)	0.000
Self-Acceptance	20.97	19.85	−1.12 (0.28)	18.34	22.59	4.24 (0.25)	5.37 (0.38)	0.000	5.37 (0.34)	0.000
**Overall Quality of life**										
Social relations	64.67	68.67	4.00 (1.26)	69.38	71.95	2.57 (1.01)	−1.42 (1.77)	0.422	−1.43 (1.80)	0.430
Living conditions	60.93	64.66	3.73 (1.44)	64.68	74.01	9.34 (0.87)	5.60 (1.69)	0.001	5.60 (1.77)	0.002
Personal independence	48.10	56.04	7.94 (2.29)	58.84	61.56	2.72 (0.89)	−5.22 (2.31)	0.024	−5.22 (1.77)	0.003
**Quality of neighbourhood social environment (%)**										
Drugs and substance abuse	8.7	7.5	−1.2 (2.8)	3.8	8.0	4.2 (2.6)	5.4 (4.2)	0.196	5.4 (3.9)	0.169
Gambling	10.0	17.4	7.4 (3.2)	11.2	17.4	6.2 (3.1)	−1.2 (4.6)	0.793	−1.2 (4.7)	0.796
Neighbourhood safety	76.6	83.9	7.3 (5.2)	89.2	89.9	0.7 (3.5)	−6.6 (5.8)	0.254	−6.6 (5.1)	0.197
**Self-esteem**										
Self Esteem	18.62	14.78	−3.84 (0.36)	21.98	22.77	0.80 (0.27)	4.64 (0.49)	0.000	4.64 (0.52)	0.000

*UDiD* Unadjusted difference-in-differences

*ADiD* Adjusted difference-in-differences

^a^ Adjusted for age, gender, years of education, living conditions, marital status, and employment status

^b^ Kernel propensity-score matching DID treatment effects with common support of the propensity scores

Overall, the qualitative insights from both FGDs and KIIs demonstrate an overwhelming impact of the program on QoL – particularly, the living conditions of beneficiaries as a result of mutual interdependence rather than personal independence (**Table** **5**). Factors that facilitated the project impact on QoL include establishing functional, successful community-owned savings and credit associations locally known as village saving and loan associations (VSLAs), and training their members in business transactions and entrepreneurship. VSLAs were self-managed groups that facilitated peer-to-peer lending without any external funding to address many social issues that disenfranchise youth from accessing mainstream banking and financial services. Savings groups were formed and constituted a platform through which members could make safe savings, access loan, and obtain emergency-loan insurance. The project allowed the training of a skilled cohort with high financial literacy and entrepreneurial skills whose members may potentially act as mentors and role models for other young people in the community. Moreover, the model would likely be sustained and replicated by successful VSLA members. This, together with demonstrated community commitment and ownership of the initiative, suggested that the VSLA’s may be self-sustaining model that will continue to provide disenfranchised youth with access to adequate resources to improve their living standards beyond the life of the project.

**Table 5:**
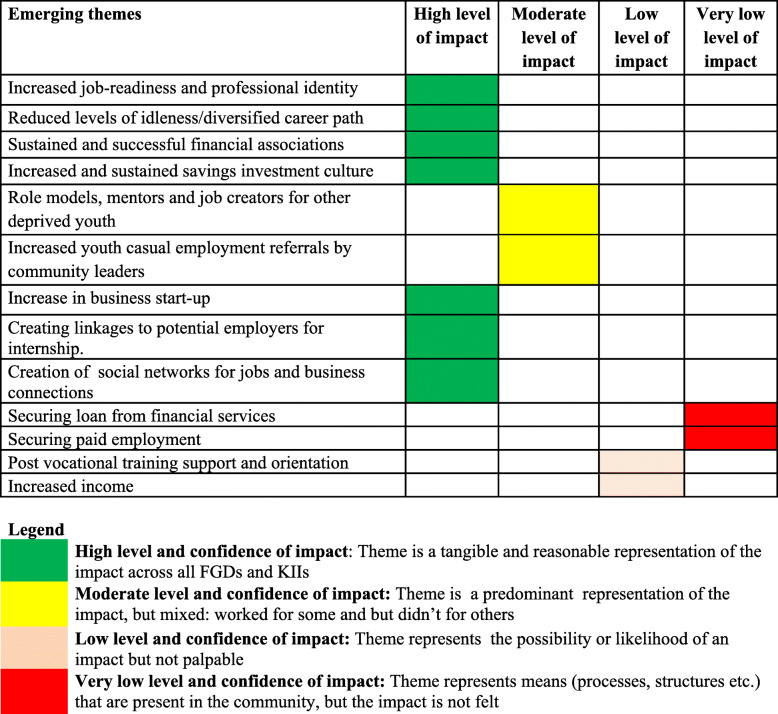
Impact of the UPLIFT project on the wellbeing and quality of life of young people in Makindye and Nakawa divisions

For example, one association recounted how it started with a base capital of only Shs. 50,000 ($US14, July 2017 exchange rate) from 20 members, and within two years the portfolio had grown to Shs. 6,300,000 (USD, 1750; July 2017 exchange rate). Many other associations recorded similar success. However, as was the case of KCCA Centre Youth Loan, only young people with existing businesses and seeking to expand, rather than those who were unemployed, enjoyed immediate benefits. This is because members of the association buy shares in the group, and each member can only buy shares based on their purchasing power or what they can afford; and the capacity to borrow is based on the number and value of shares each association member has. Some project beneficiaries recounted their personal stories and noted:

“*The saving group has helped me improve my grocery business and also see it move forward and prosper. From the money I have got from my business, am going to start a poultry business as a way of diversification. I use the money from my grocery business to get my basic needs. I had my business even before joining the saving group but I got money from this group to expand my business, before I could only be able to buy a small container of goods but am now able to buy in larger quantities*”, FGD, women VSLA

“*Since I joined this saving group, I have realised that it can help improve one’s standards of living….since I joined this group two years back, I have been able to upgrade from being a bicycle rider to a motorcycle rider. Some of us can’t get loans from the bank but can be able to borrow money from this group and better my life without any worries of my property being confiscated. I have also been able to build a better home for myself so generally my life has really improved since I joined this savings group*” FGD, CVA.

### The impact of the UPLIFT on psychological wellbeing

The project’s impact on the psychological wellbeing was most profound. The difference-in-difference estimation (**see** Table 4) shows that the UPLIFT project led to a 5.4-point increase in self-acceptance [DiD = 5.37 (0.34), *p* < 0.001], a 5.3-point increase in purpose in life [DiD = 5.33 (0.45)p < 0.001), a 5.7-point increase in personal growth [DiD = 5.65 (0.50)p < 0.001], and a 10.7-point increase in autonomy (DiD = 10.73 (0.40), *p* < 0.000]. However, the project had a negative impact on positive relationships (DiD = − 5.43 (0.44), p < 0.001]; and no observable impact on environmental mastery (DiD = − 0.72 (0.49), *p* = 0.141]. Some of the emerging themes from the qualitative data that support the impact of the intervention on psychological wellbeing are summarised in **Tables** **5****&** **6**. VSLAs particularly benefited women and there was also an acknowledgement that women fared better than men in terms of employment following vocational training. Factors that facilitated the intervention’s impact on psychological wellbeing included putting in place effective and strengthened systems for reporting crimes; hope for the future among girls (some of whom have become community leaders, role model mothers, and ambassadors of change); establishing an effective and sustainable platform for facilitating the participation and involvement of youth in decision making; reduced criminal activities within the community (with notorious young gang groups like the KIFEESI and UNATO weakened as some of their members were identified, trained and counselled, which gave them a different successful career path); putting in place an effective and sustainable platform for facilitating the participation and involvement of youth in decision making; training a skilled cohort with strong functional skills, high financial literacy and shrewd entrepreneurial skills whose members will continue act as mentors and role models for other young people in the community; and forming, coordinating, and training functional committees and community structures including social networks, child protection committees, civic engagement committees, and disability councils. These community structures and training platforms have engendered an informed, vibrant, and empowered youth full of confidence and self-esteem knowing to get what they want.s

**Table 6:**
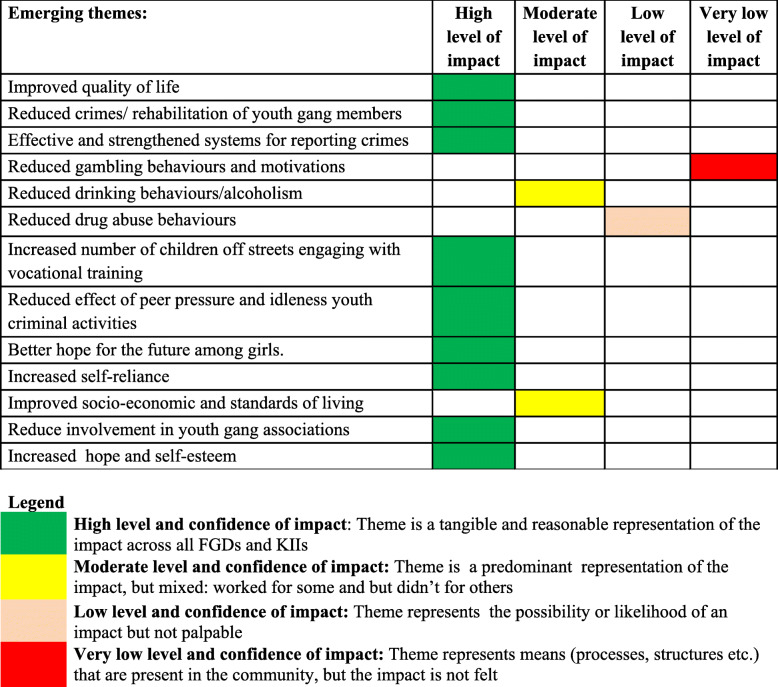
Overall findings from qualitative data on the project’s impact on job market and employment

As participants explained:

*The project has taught me how to keep my money but now I know that for each little money I get I have to save with this group so that in future when it’s time for me to start my own business*. *I have learnt time management since I know we are supposed to show up for the savings group meetings on time. I have also been able to make friends from the saving group, which creates trust and cohesion among members. I will have the capital I need to start a business. I want to get my own general merchandise shop. Our association members will help me achieve this dream, learning from them and getting their support and mentoring.* FGD, women VSLA

*We have been taught how to start up our own businesses with any amount of money one has. One of our members was able to start up her own business with only shs.50,000 and now her business is worth shs.300,000 in less than 2 years so her business is continuously expanding so we are very grateful for this*. FGD, CVA market

In addition, members who did not have businesses prior to joining the associations benefited in other ways. Most of the associations have welfare accounts to assist members during emergencies such as funeral costs, school fees, or other unexpected expenses. The associations also allow members to build capital to start a business in the future. Participants noted:

*I have learnt to use my money appropriately since I know I have to have money every Wednesday for the saving group and I will yield later in the future. When I need money for my children’s school fees, I know I can be able to get a soft loan from this savings group and pay back at lighter interest rates. The interest rate is five per cent and we usually give someone a month to pay back and when one is unable to pay back within a month, we give them an additional two months but for each extra month, another five per cent interest rate applies.* FGD, Youth Group.

### The impact of the UPLIFT on self-esteem and quality of the neighbourhood social environment

In Table 4, the difference-in-difference estimation of the project’s impact on self-esteem shows that there was a 4.6-points increase in self-esteem [DiD = 4.64 (0.52), *p* < 0.001]. This finding is supported by themes emerging from the qualitative component (Table 6) and is consistent with the interventions’ design theory of change (Table 2). Young people completing the courses and successfully gaining casual employment or establishing a business reported a boost in their self-esteem and instilled a sense of pride in being able to contribute to meeting their family needs. As participants explained:

*We have a group of young people in our saving group who now have jobs and we have recommended some of them …Through these savings, some people have created jobs and have employed other people which has helped to reduce the problem of unemployment.* FGD, Youth Group.

*Before the project, I had to ask my guardian for everything like toothpaste, toothbrush, food, drinks… but now I am also able to buy food for her because I am now working and can also sustain myself.* KII, Project Partner.

There was no observable impact on the quality of neighbourhood social environment, including drug and substance use, gambling and neighbourhood safety (Table 4). This finding is consistent with insights from the qualitative data. Despite the project establishing linkages to potential employers and social networks to facilitate internships, employment opportunities and business connections, it had no observable impact on subsequent paid employment or post vocational training support and orientation. After completing the vocational training, young people still struggled to find paid work, often being mistrusted by potential employers due to their past backgrounds and/or reputation and their lack of experience. Frequently, those who could gain employment upon completion of the course reverted to unhealthy lifestyles when they felt underpaid or exploited. As some participants stated:

*[D]espite the training and the support we get from the UPLIFT project, our backgrounds always haunt us in the face of a potential employers… drinking, drug abuse, and too much HIV/AIDS, …since we cannot feed well so we can’t be able to work. Then there is gambling. If we had had activities to engage in like carpentry, welding there would be minimal anti-social behaviour like alcohol and drug abuse after graduation…*FGD, Youth Group.

*Gambling remains one of the main threats to young people completing the course. It is a major problem to many youth... where I stay, there are five sports betting centres so when one wakes up, have no job to go to, they go straight to the betting centres instead of looking for jobs. In the long run, some youth who have nothing to do eventually resume criminal activities. KII, Project Partner.*

As indicated earlier the project had an impact on youth criminal activities in the community, whereby gang groups like the KIFEESI and UNATO had been weakened as some of their members were identified, trained and counselled. Such training put them in different career paths. Participants’ voices affirmed the reduced effect of peer pressure and idleness that led to youth criminal activities as well as the increased number of youth off streets engaging with the vocational and other training activities, However, FGD participants and key informants reiterated that drug use and gambling remain serious issues that may continue to impact negatively on the achievements made by the UPLIFT project in the long term. Although the UPLIFT project attempted to work with gambling houses, the effort was fruitless because gambling house owners have considered the project as a potential threat to their businesses. Besides, there is no by-law instituted to control the activities in gambling places. Nevertheless, through the social accountability activities, the project engaged local leaders and the process of developing this by law has already started. As they summed it up:

*Alcohol and drug abuse are continuing to affect our communities. There is no doubt that the UPLIFT has reached out to the youth through radio talk shows. We also have a centre for the youth here in Banda where the youth are being rehabilitated and sensitized to encourage them to change behaviours. However youth are still engaging in drugs and alcoholism, some engage in these activities due to peer pressure, for personal pleasure, and most importantly for lack of opportunities after their vocational training.* FGD, CVA.

*Some youth have no feasible alternative sources of income so they engage in gambling to make money to be able to save in the savings groups. The desire to save sometimes has facilitated some youth to engage in gambling to be able to save*. KII, KCCA.

## Discussion

Our QoL comprised three main variables: social relations, living conditions, and personal independence. The findings show that the UPLIFT project has resulted in improved living conditions. The positive impact observed for living conditions is consistent with similar studies in slums of neighbouring Kenya and in Niger [51, 52]. The observed improvement in living conditions were mainly associated with improved financial conditions where participants were able to acquire capital through their associations (a form of social capital). The capital provided opportunities to expand business, which translated into significant financial gain; and the improved financial conditions resulted in increased living conditions such as being able to provide daily necessities of life, housing, food, and health related outcomes. The relationship between living conditions and income has been highlighted by some researchers who studied the impact of mobile money transfers on living conditions in sub Saharan Africa [51]. We argue that the observed improvement in the living conditions of intervention participants is moderated by an improvement in their financial circumstances as a result of the UPLIFT intervention. This argument challenges a previous investigation of living conditions among slum dwellers in Dakar, Senegal and Nairobi Kenya, which concluded that the level of education, jobs and income were not linked to improved living conditions [53].

The negative impacts of the intervention on personal independence must be interpreted with caution because many factors come into play. Young people became dependent on assistance in many daily life situations, which eroded their ability to maintain a sense of independence and increased their sense of uncertainty. They belonged to informal savings associations and they depended on these associations for support and micro-loans. Given that most participants in the intervention are rural migrants living with relatives or nonrelatives with whom they do not get along, there are limited opportunities for family and other social connections; such an environment creates the atmosphere of mistrust, and other risks associated with in the urban peripherals as espoused by an earlier study of slum life in Nairobi city [54].

There was a profound improvement in young people’s psychological wellbeing and self-esteem. It is worth noting that self-esteem is closely related to psychological well-being. Forming, coordinating, and training functional committees and community structures, including social networks, child protection committees, civic engagement committees and disability councils represented platforms to maximise young people’s socio-economic participation; and engendered an informed, vibrant, empowered youth exhibiting confidence and knowing where, when, and how to get what they want. Young people’s increased self-confidence and evaluation of their own worth translated into improved psychological wellbeing as the increased self-acceptance or sense of pride, dignity and hopefulness empower them with more decision-making abilities (e.g. peer-to-peer lending without any external funding), to socially and economically engage in established social networks (e.g. VSLAs) and participate in public life (e.g. volunteering for community committees). This finding is consistent with existing literature on interventions to improve psychological wellbeing of young people in low resource settings [19, 55–57].

Despite the profound positive impact on four domains of psychological wellbeing (autonomy, personal growth, purpose in life, and self-acceptance), we found negative impacts of the intervention on positive relationships but no observable impact on environmental mastery. The negative impact on positive relations and lack of observable impact on environmental mastery depict outstanding gaps requiring tailored interventions to enhance wellbeing among the youth; positive relationships and environmental mastery are intrinsically linked to  psychological wellbeing. Literature suggests that positive relations are outcomes of behavioural choices that contribute to individual work efforts and productivity [58]. Addressing positive relations in our study context will elicit the desired behaviour and build trust with potential employers and the communities at large to overcome the daunting past experiences, antisocial behaviour and backgrounds that limit young people’s opportunities. Similarly, an intervention to address environmental mastery is an important aspect that would emphasise the need for youth to develop and harness abilities control their surrounding context and events so as to manage everyday affairs and take advantage of opportunities that abound therein [59].

Our findings mirror studies reporting the effects of financial capabilities and social protection programs on wellbeing across sub Saharan Africa [60–62]. The financial capability described in the literature is similar to VSLAs, the most important aspect of the intervention to build young people’s psychological wellbeing in slum areas. It encompasses people’s financial knowledge and management skills to understand their own financial circumstances, and to be in control of their money regardless of their income levels  [63, 64]. This requires young people to manage their money and live within their means, to plan ahead to cope with unexpected events and making decision for the long run, to be aware of available financial services and making right choices to access them, and to get help to get things right [63]. For example, financial difficulties have been found to explain 7% of variance in life satisfaction, 10% of the variance in self-esteem, 10% of variance in locus of control, and 9% of the variance in depression among the poor groups of England [64]. Shifting individuals with relatively low levels of financial capability to an average level of capability account for 6% improvement in their psychological wellbeing [63]. However, the impact is strongest at the bottom of the financial incapability distribution [63], as would be the case of young people in slum areas in our study context.

The initiation and strengthening of the VSLAs through existing community structures and assets meant that various committees bringing young people into interaction with other key members of the communities were put in place. These VSLAs and established community committees enhanced young people’s socio-economic participation as these initiatives adopted a non-political approach and embraced disenfranchised youth in the target communities. Political commitment and good local government meant that the intervention worked with existing government structures including the police, the education structure (primary and secondary schools), the local council structures, the justice system, and community-owned youth associations, and service providers. This partnership allowed UPLIFT to reach school drop outs; victims of violence, sexual exploitation, commercial sex work, and child labour; and youth caught up in drug abuse and gambling to address their needs through capacity building and vocational training.

Therefore, with a good co-operation within the community, framing the socio-economic and citizen participation through voluntary groups maximised the participation and involvement of key community stakeholders, local leaders, UPLIFT targeted constituency and KCCA officials. The existence of functional community-owned committees, community structures to meet the capacity building and training needs of community members, and the incorporation of emergency-loan insurance in VSLAs as well as the multiplication and replication of the model are hallmarks of demonstrated community commitment and ownership of the initiative. Therefore, harnessing these networks gave VSLAs a strong structure and base through which disenfranchised youth had access to loans and associated financial literacy and business training to fight poverty. Access to adequate financial resources to improve young people’s living standards gave them a sense of hopefulness and long term purpose of life, an increased sense of worth and the awareness of their strengths and weaknesses to run a business or participate in community initiatives (self-acceptance), a sense of determinism and independence as well the ability to resist social pressures such as peer pressure to re/join criminal groups or to adopt unhealthy behaviours (autonomy), and opportunities for new experiences and ongoing learning (personal growth) [55, 65].

Although current evidence suggests that increased autonomy and self-acceptance as a result of improved availability of financial resources and associated financial literacy and business training translate into increased self-respect and reduced shame, leading to positive relations, UPLIFT had a negative impact on positive relationships. This finding is closely related to the negative impacts of the intervention on personal independence and social relations discussed earlier and can be attributed to contextual factors including being a rural-urban migrant living in slums, limited trust of the surrounding risks associated with living in slums.

We argue that UPLIF had no impact on environmental mastery, a finding that could be closely related to the slum environment and the fact that the majority of slum dwellers are rural-to urban migrants. For example, key and successful areas of socio-economic participation achieved by the project included increasing the communities’ ability to have a level of control of socio-economic issues important young people; employment and social participation; income, assets and wealth creation through VSLAs; socio-political and civic rights and participation; and community capital, support, and networks. However, the slum environment is too complex for your people to manipulate, manage, and master, which impedes their ability to select, shape, and achieve circumstances and environments that match their goals and career paths. Environmental mastery is a critical aspect of psychological wellbeing [66]; therefore, inability to master the environment undermines efforts to improve youth wellbeing. This is consistent with literature from other parts of Africa and beyond suggesting inability to harness and control environment has an inverse relationship with wellbeing and reduces mindfulness [67, 68].

Our data suggest that UPLIFT had no observable impact of the quality of neighbourhood social environment. This finding could be related to the environmental mastery discussed above. It could also be due to the fact that items of this construct were measured on a “yes” or “no” scale. For example, the single item measuring gambling has been found to be unable to discriminate among respondents with varying levels of the trait problem gambling, and hence not a reliable measure of at-risk problem gambling [69]. However, the advantage of the single item question on gambling is its simplicity to be used in complex interventions at the expense of more useful and detail information. Evidence suggests that single items have relative disadvantage to multi-item measures because more items are less prone to distortion and provide replies that are more consistent [70]. The self-report nature of the drugs and substance abuse and the social cultural dimensions of slum environment characterised by the perceived social  and self-stigma, in people’s willing to reveal their drugs and substance abuse activities means that measuring this concept with accuracy remains a challenge. Further studies are needed to develop and validate good measures of quality of neighbourhood social environment for use in interventions.

## Conclusion

This research examined the impact of the UPLFIT project on quality of life, psychological wellbeing, self-esteem, and the quality of neighbourhood social environment of the youth from the slums of Kampala, Uganda. The findings show that the project contributed to improvements in the quality of life, psychological wellbeing, and self-esteem outcomes, without the same for the neighbourhood social environment outcomes. The findings also highlight the significant role of micro finance through community-owned assets accumulation and capacity building initiatives for young people in slum areas for improving critical QoL and psychological wellbeing outcomes.

Overall, our research demonstrates the need for evidence based interventions to optimise resource allocation for impact and the need to understand contextual issues for targeted interventions. Where no observable impact was realised, calls for the need to further investigate the appropriateness of interventions and innovations that can produce favourable outcomes. In addition, in the context of Uganda, there is urgent need for targeted evidence based interventions that will yield the intended objectives and save the scarce resources from non- impactful interventions, while harnessing Uganda’s demographic dividend. The Ugandan government may put in place a policy that mandates interventions aiming to improve young people’s QoL to have strong and measurable outcomes. Such a policy will affect impact reporting; thus capturing the quantification of investments and contribution to Uganda’s largest proportion of the population, the transformation to the middle income country, and better reporting toward the SDGs. Such reporting will also have cascading effects on improving social relations and accelerate the impetus for a hopeful nation with better QoL, and wellbeing.

Our research demonstrates that measuring of QoL interventions in low resource settings is of utmost importance for evidence-based decision making. Therefore, we anticipate that this study will accentuate the need for governments and aid agencies to measure the impacts of their QoL interventions.

## Data Availability

The datasets generated during and/or analysed during the current study are not publicly available due to World Vision’s standing agreements with partners to protect the privacy of intervention beneficiary communities. Data are available from the corresponding author upon request for researchers who meet the criteria for access to confidential data.

## Abbreviations

BSLA: Bussiness Savings and Loan Association
CBT: Community Based Trainers
CVA: Citizens Voice and Action
CPC: Child protection Committe
FGD: Focus Group Discussion
HIV: Human Immunodefficiency
KCCA: Kampala Capital City Authority
KIIs: Key informant interview
LMICs: Low and Middle-Income Countries
MDG: Millennium Development Goals
NAADS: National Agricultural Advisory Service
OWC: Operation Wealth Creation
QoL: Quality of Life
SAACOs: Savings and Credit Co-operatives
SRH: Sexual Reproductive
SRHR: Sexual and Reproductive Health Rights
SDG: Sustainable Development Goals
UPLIFT: Urban Program on Livelihoods and Income Fortification and Socio-civic Transformation
USD: United States Dollars
VSLA: Village Saving and Loan Association
WHOQOL-BREF: World Health Organization Quality of Life Instrument
